# Draft Genome Sequence of *Bacillus* sp. GZT, a 2,4,6-Tribromophenol-Degrading Strain Isolated from the River Sludge of an Electronic Waste-Dismantling Region

**DOI:** 10.1128/genomeA.00474-16

**Published:** 2016-06-02

**Authors:** Zhishu Liang, Guiying Li, Taicheng An, Ranjit Das

**Affiliations:** a State Key Laboratory of Organic Geochemistry and Guangdong Key Laboratory of Environmental Protection and Resources Utilization, Guangzhou Institute of Geochemistry, Chinese Academy of Sciences, Guangzhou, China; b Institute of Environmental Health and Pollution Control, School of Environmental Science and Engineering, Guangdong University of Technology, Guangzhou, China; c University of Chinese Academy of Sciences, Beijing, China

## Abstract

Here, we report the draft genome sequence of *Bacillus* sp. strain GZT, a 2,4,6-tribromophenol (TBP)-degrading bacterium previously isolated from an electronic waste-dismantling region. The draft genome sequence is 5.18 Mb and has a G+C content of 35.1%. This is the first genome report of a brominated flame retardant-degrading strain.

## GENOME ANNOUNCEMENT

TBP (2,4,6-tribromophenol), a widely used brominated flame retardant, is released into the environment, resulting in deleterious effects on the environment and human beings, triggering effective techniques for its removal (1, 2). Among the techniques used, biodegradation has been considered an important means to bioremediate TBP-polluted sites due to its various merits (3). To date, only several papers have studied the biotic transformation of TBP using activated sludge or isolated strains (4). *Bacillus* sp. strain GZT, capable of efficient biodegradation of TBP, was isolated from a creek near electronic waste-dismantling workshops using TBP as the sole carbon and energy source (5). The genome of GZT was sequenced to gain insight into the TBP degradation mechanisms and the unique physiological and genetic features of the species within the genus *Bacillus*. This work can potentially improve the effective control of TBP pollution in future practice.

The genomic DNA of GZT was extracted using the Wizard genomic DNA purification kit (Promega, USA), per the manufacturer’s instructions, and quantified by use of a NanoDrop 2000. The genome sequencing was performed using an Illumina MiSeq system (Shanghai Majorbio Bio-pharm Technology Co., Ltd., China). A total of 2,196,615 paired-end reads, with an average insert size of 400 bp, were generated by sequencing, which yielded approximately 208-fold depth of coverage. The reads obtained were assembled using SOAP*denovo* version 2.04 (6), and the optimal k-mer of 31 was selected by ABySS version 1.3.7 (7). The final assembly consisted of 81 scaffolds with a total size of 5,188,803 bp and a G+C content of 35.1%. The scaffolds consisted of 96 contigs of 5,188,788 bp, with an *N*_50_ of 166,003 bp, and the longest contig was 499,335 bp.

The draft genome of GZT was analyzed using the NCBI Prokaryotic Genome Annotation Pipeline (PGAP) (8). Genome annotation was done using the RAST server with the SEED database (9). A total of 5,581 putative open reading frames (with an average size of 791 bp), 5,252 protein-coding sequences (CDSs), 127 pseudogenes, and 52 RNA-coding genes (with 36 tRNAs, 11 rRNAs, and 5 noncoding RNAs [ncRNAs]) were identified. The genome sequences in the RAST database showed that the closest neighbors of GZT were *Bacillus cereus* AND1407 (score, 537) and *B. cereus* ISP3191 (score, 514).

Six dehalogenase subunits were found in the genome of GZT, including the previously confirmed haloacid-degrading gene cluster in *B. cereus* and *Bacillus thuringiensis*. However, which dehalogenase in GZT is directly related to the TBP degradation is still unknown and needs further identification. The genome information and annotation reported here are valuable for future research aimed at better understanding the molecular principles of TBP degradation.

### Nucleotide sequence accession numbers.

This draft genome sequence of *Bacillus* sp. GZT has been deposited at DDBJ/EMBL/GenBank under the accession no. LVVJ00000000. The version described in this paper is the first version, LVVJ01000001.
